# Supplementary material for: Genomic insights unveil the plasmid transfer mechanism and epidemiology of hypervirulent Klebsiella pneumoniae in Vietnam
Source: Nat Commun. 2024 May 17;15:4187. doi: 10.1038/s41467-024-48206-3 (PMC11101633; doi:10.1038/s41467-024-48206-3)
Supplement: Supplementary file 1 — Supplementary Information [file 41467_2024_48206_MOESM1_ESM.pdf]

SUPPLEMENTARY INFORMATION

Table S1: Primer sequences for the detection of four virulence genes in *Klebsiella pneumoniae* by qPCR

Table S2: Characteristics of five publicly available *iuc3*-carrying plasmids from hypervirulent *K. pneumoniae* isolates for comparisons with *iuc3*-carrying plasmids identified in this study

Figure S1: The annual distribution of bloodstream infections caused by K. pneumoniae (red), hypervirulent K. pneumoniae (green) and hypervirulent K. pneumoniae ST23 (blue)

Figure S2: Melting curve analysis illustrates the melting temperature peaks (Tm) of four target virulence genes (*iucA*, *iroB*, *rmpA and rmpA2*) in multiplex PCR assay

Figure S3: Phylogenetic structure of hypervirulent *Klebsiella pneumoniae* ST23 isolates from Vietnam in a global context

Figure S4: BLAST comparisons of three *iuc3* plasmid sequences (pQY1, pQY2, pQY3) identified in this study together with five publicly available *iuc3* plasmids

Table S1: Primer sequences for the detection of four virulence genes in *Klebsiella pneumoniae* by qPCR

Gene	Primer sequence (5'-3')	Primer size (bp)	Tm of primer	Tm of amplicon
iucA-F2	GCATCTCAACCTGCAACAAC	164 bp	63.4	91.5
iucA-R2	TCAGCCCTTTAGCGACAAG		63.1	
iroB-F2	GCAGCAGAGGCGGGATTG	172 bp	69.5	92
iroB-R3	TGGAATACCGGCGTGTAGC		65.9	
rmpA-F4	GCAATGCCTACTGAAAAATGG	179		
rmpA-R3	GCATGAGCCATCTTTCATCAAC			
rmpA2-F2	TTAAAAAGCCGTCTAAAAATTG	152 bp	58.9	79
rmpA2-R2	GTATTTGATGTGCACCATTT		57.3	

Table S2: Characteristics of five publicly available *iuc3*-carrying plasmids from hypervirulent *K. pneumoniae* isolates for comparisons with *iuc3*-carrying plasmids identified in this study

Plasmid name	Accession number	Size	Country	Host	Collection date	Sequence Type	Virulence genes	AMR genes
p16114547	MK649829	187989	Laos	Human	2016	ST290	iuc3	qnrS1, *tetA* , *bla* TEM-*30*
p130411–38618	MK649826	241799	Vietnam	Human	2011	ST17	iuc3	strAB, *aadA1* , *cmlA5* , *floR* , *arr2* , *sulII* , *tetA* , *dfrA14* , *bla* OX[illegible]
KPCTRSRTH01	CP041094	168755	Thailand	Pig	2016	ST3541	iuc3	-
CHS 43	NZ_KK737127.1	225357	United States	Human	2013	ST25	iuc3	-
CHS 48	NZ_KK737178.1	223874	United States	Human	2013	ST25	iuc3	-

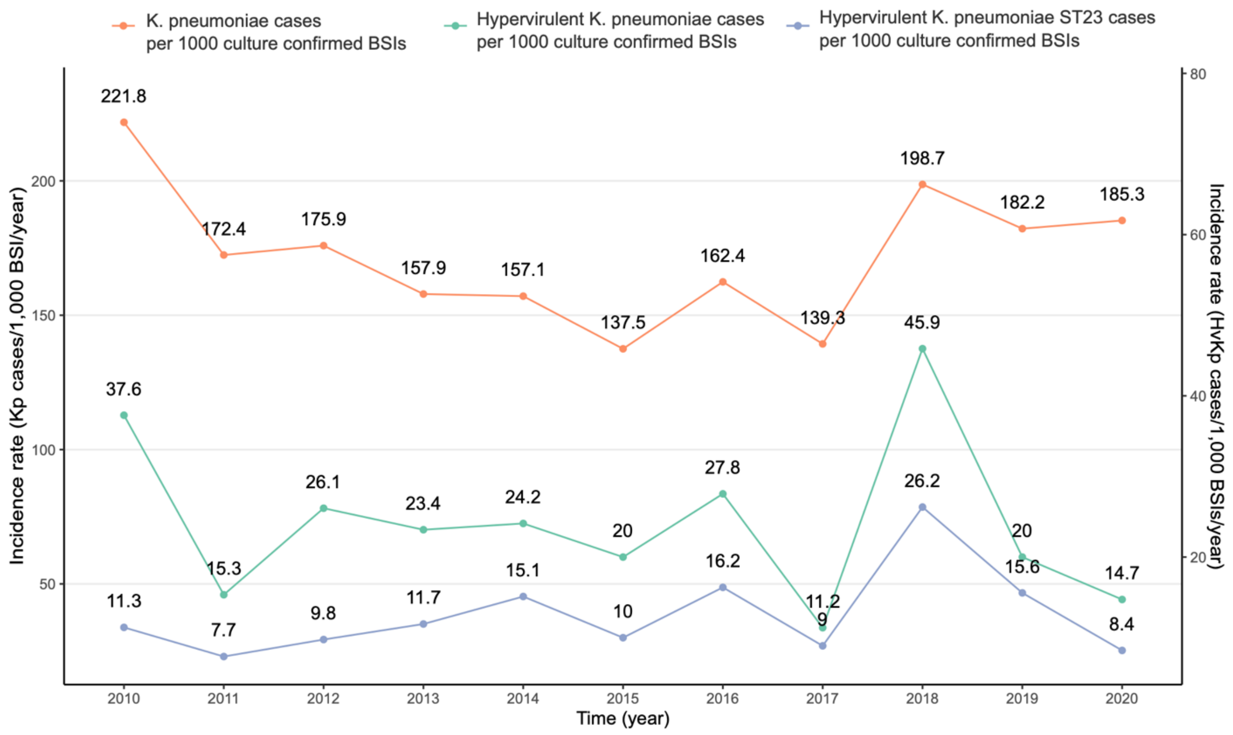

Figure S1: The annual distribution of bloodstream infections caused by *K. pneumoniae* (red), hypervirulent *K. pneumoniae* (green) and hypervirulent *K. pneumoniae* ST23 (blue)

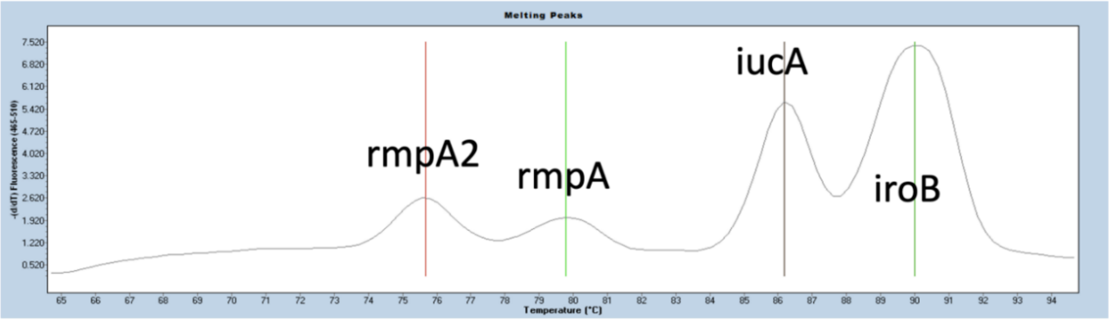

Figure S2: Melting curve analysis illustrates the melting temperature peaks (Tm) of four target virulence genes (*iucA, iroB, rmpA and rmpA2*) in multiplex PCR assay.

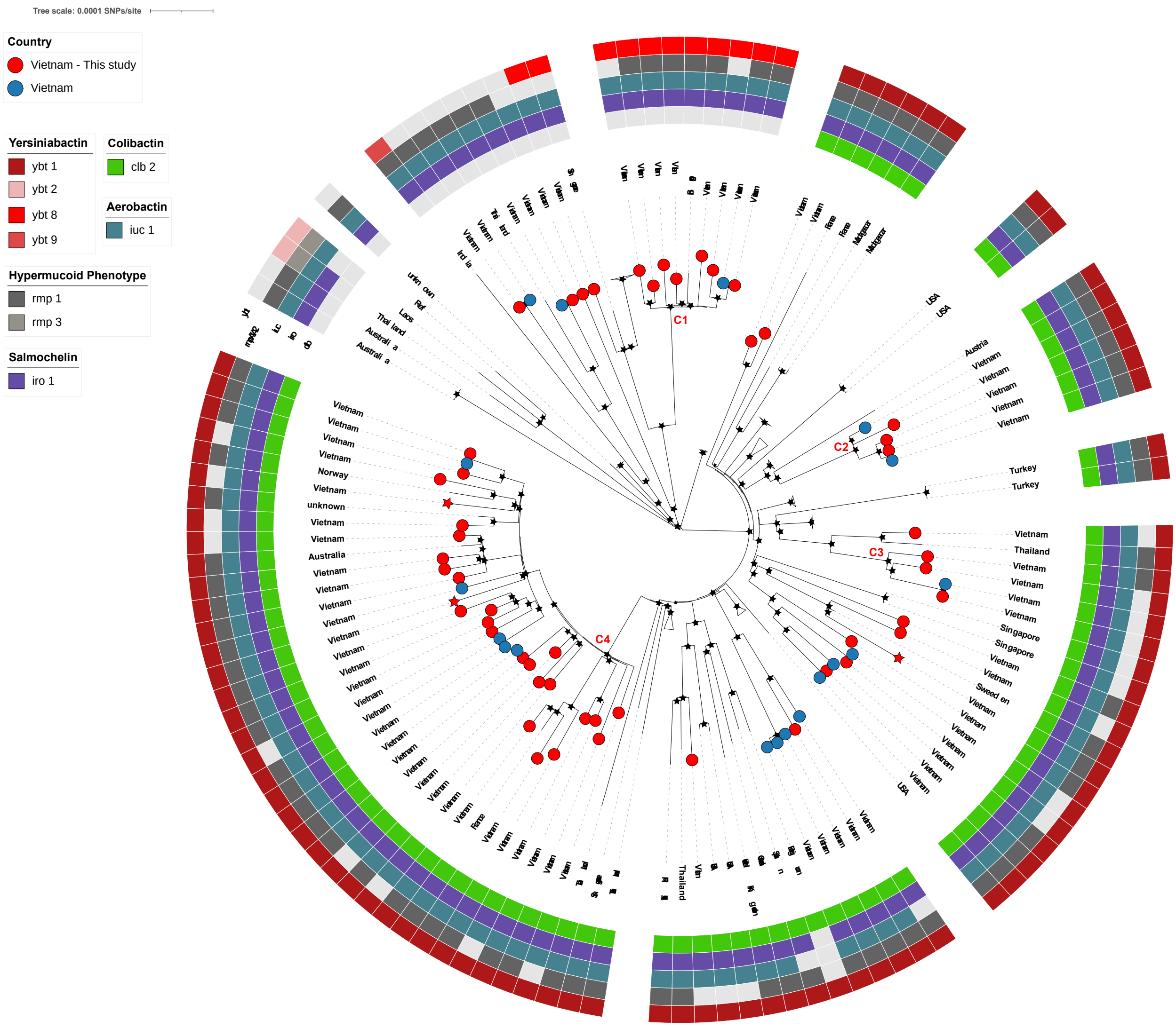

Figure S3: Phylogenetic structure of hypervirulent *Klebsiella pneumoniae* ST23 isolates from Vietnam in a global context.

The phylogenetic tree is mid-point rooted. hvKp ST23 isolates in Vietnam are highlighted by the colored circles at the terminal nodes of the tree. The tip labels indicate the country of isolation. The tree scale bar represents the number of SNPs per site. The black stars highlight bootstrap support values ≥80% on internal nodes, with larger stars indicating higher bootstrap values. The heat map displays the presence (in color) or absence (in grey) of different lineages of virulence loci encoding yersiniabactin, colibactin, salmochelin, aerobactin and hypermucoid phenotype. Source data for the heat map are provided as a Source Data file.

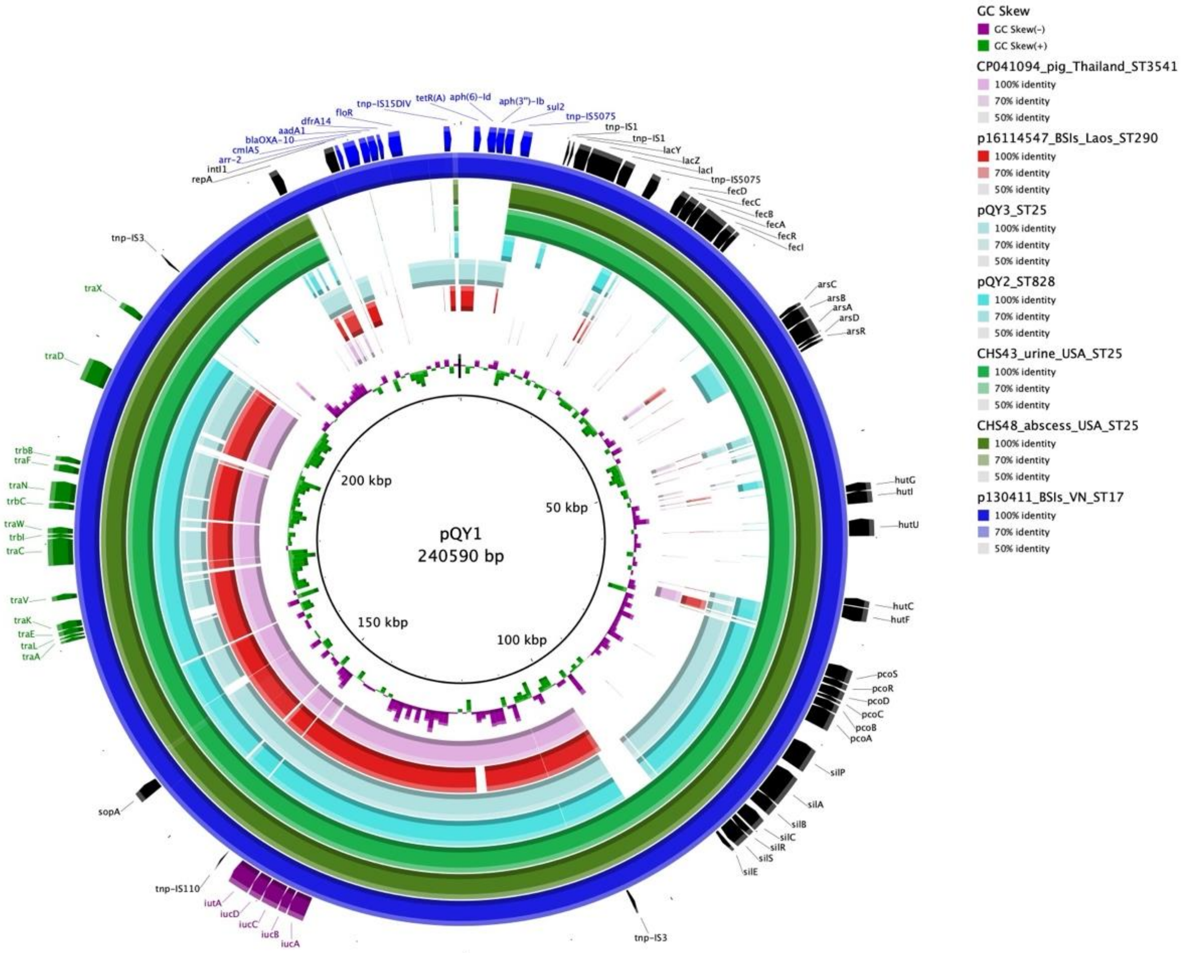

Figure S4: BLAST comparisons of three *iuc3* plasmid sequences (pQY1, pQY2, pQY3) identified in this study together with five publicly available *iuc3* plasmids

The reference sequence of pQY1 (FIBK:FII – ST25) is shown by the central circle. The GC skew of reference plasmid is shown in the innermost ring. The remaining inner rings show the nucleotide similarity between the reference plasmid pQY1 and other *iuc3* plasmids from this study (pQY2_ST828 and pQY3_ST25) as well as the five *iuc3* plasmids previously characterized in hvKp isolates (CP041094_pig_Thailand_ST3541, p16114547_BSIs_Laos_ST290, CHS43_urine_USA_ST25, CHS48_abscess_USA_ST25, p130411_BSIs_VN_ST17). The outermost ring displays gene annotations of the reference plasmid, emphasizing the shared virulence module in purple, the conjugation module in green, and the antibiotic resistance module in blue.
