# Supplementary material for: Genomic insights unveil the plasmid transfer mechanism and epidemiology of hypervirulent Klebsiella pneumoniae in Vietnam
Source: Nat Commun. 2024 May 17;15:4187. doi: 10.1038/s41467-024-48206-3 (PMC11101633; doi:10.1038/s41467-024-48206-3)
Supplement: Supplementary file 3 — Description of Additional Supplementary Files [file 41467_2024_48206_MOESM3_ESM.pdf]

Description of Additional Supplementary Files

Supplementary Data 1: Metadata information and accession numbers of 176 global hypervirulent K. pneumoniae ST23 isolates
