# Supplementary material for: Genomic insights unveil the plasmid transfer mechanism and epidemiology of hypervirulent Klebsiella pneumoniae in Vietnam
Source: Nat Commun. 2024 May 17;15:4187. doi: 10.1038/s41467-024-48206-3 (PMC11101633; doi:10.1038/s41467-024-48206-3)
Supplement: Supplementary file 5 — Reporting Summary [file 41467_2024_48206_MOESM5_ESM.pdf]

nature portfolio

Corresponding author(s): Pham Thanh Duy

Last updated by author(s): 02 April 2024

Reporting Summary

Nature Portfolio wishes to improve the reproducibility of the work that we publish. This form provides structure for consistency and transparency in reporting. For further information on Nature Portfolio policies, see our Editorial Policies and the Editorial Policy Checklist.

Statistics

For all statistical analyses, confirm that the following items are present in the figure legend, table legend, main text, or Methods section.

n/a	Confirmed	
☐	☒	The exact sample size (*n*) for each experimental group/condition, given as a discrete number and unit of measurement
☐	☒	A statement on whether measurements were taken from distinct samples or whether the same sample was measured repeatedly
☒	☐	The statistical test(s) used AND whether they are one- or two-sided<br>*Only common tests should be described solely by name; describe more complex techniques in the Methods section.*
☒	☐	A description of all covariates tested
☒	☐	A description of any assumptions or corrections, such as tests of normality and adjustment for multiple comparisons
☒	☐	A full description of the statistical parameters including central tendency (e.g. means) or other basic estimates (e.g. regression coefficient) AND variation (e.g. standard deviation) or associated estimates of uncertainty (e.g. confidence intervals)
☒	☐	For null hypothesis testing, the test statistic (e.g. *F*, *t*, *r*) with confidence intervals, effect sizes, degrees of freedom and *P* value noted<br>*Give P values as exact values whenever suitable.*
☒	☐	For Bayesian analysis, information on the choice of priors and Markov chain Monte Carlo settings
☒	☐	For hierarchical and complex designs, identification of the appropriate level for tests and full reporting of outcomes
☒	☐	Estimates of effect sizes (e.g. Cohen's *d*, Pearson's *r*), indicating how they were calculated

Our web collection on statistics for biologists contains articles on many of the points above.

Software and code

Policy information about availability of computer code

Data collection	Raw Illumina reads of publicly available ST23 genomes were downloaded from the Enterobase using the ENAdatabase-Downloader script (https://github.com/chauvinhtth13/ENAdatabase-Downloader).
Data analysis	All software utilized for data analysis is open source.

For manuscripts utilizing custom algorithms or software that are central to the research but not yet described in published literature, software must be made available to editors and reviewers. We strongly encourage code deposition in a community repository (e.g. GitHub). See the Nature Portfolio guidelines for submitting code & software for further information.

Data

Policy information about availability of data

All manuscripts must include a data availability statement. This statement should provide the following information, where applicable:

- Accession codes, unique identifiers, or web links for publicly available datasets
- A description of any restrictions on data availability
- For clinical datasets or third party data, please ensure that the statement adheres to our policy

All data used in this work are publicly available in the European Nucleotide Archive (ENA) under project numbers PRJEB64408 (https://www.ebi.ac.uk/ena/browser/view/PRJEB64408) and PRJEB26814 (https://www.ebi.ac.uk/ena/browser/view/ PRJEB26814). Source data are provided with the paper. There is no restrictions on data availability.

Research involving human participants, their data, or biological material

Policy information about studies with human participants or human data. See also policy information about sex, gender (identity/presentation), and sexual orientation and race, ethnicity and racism.

Reporting on sex and gender	This information is not reported in the manuscript
Reporting on race, ethnicity, or other socially relevant groupings	This information is not reported in the manuscript
Population characteristics	All patients with bloodstream infections aged > 25 years old.
Recruitment	This is a retrospective study. There was no new recruitment.
Ethics oversight	This study has been granted ethics approval from HTD's Ethic Committee (approval number: 1711/QĐ-BVBNĐ).<br><br>The rectal swabs/stools were sourced from two cohort studies. The first cohort involved 500 healthy children in Ho Chi Minh City, Vietnam between 2014 and 2016 to longitudinally assess the incidence rate of dysentery. Ethical approval for this study was granted by the Oxford University Tropical Research Ethics Committee (OxTREC No. 1058-13). The second cohort included healthy farmers in Dong Thap province (125 km WSW of HCMC) to assess the prevalence of AMR carriage before and after an intervention consisting of removing antimicrobial use in chicken farms. A total of 350 rectal swabs were collected from farm households during 2020. All the samples originated from healthy adults. The study received ethics approval from the Oxford University Tropical Research Ethics Committee (OxTREC No. 503-20).

Note that full information on the approval of the study protocol must also be provided in the manuscript.

Field-specific reporting

Please select the one below that is the best fit for your research. If you are not sure, read the appropriate sections before making your selection.

☒ Life sciences ☐ Behavioural & social sciences ☐ Ecological, evolutionary & environmental sciences

For a reference copy of the document with all sections, see nature.com/documents/nr-reporting-summary-flat.pdf

Life sciences study design

All studies must disclose on these points even when the disclosure is negative.

Sample size	The study did not employ sample size calculation. Instead, it conducted a retrospective analysis of all hypervirulent Klebsiella pneumoniae isolates causing bloodstream infections at a tertiary infectious diseases hospital from 2010 to 2020. Additionally, screening for hypervirulent Klebsiella pneumoniae isolates was carried out in two cohorts of healthy individuals from previous studies. While there is no available data on the carriage rate in Vietnam, the prevalence of 4.9% to 9.8% in other Asian countries justified the number of healthy individuals in each cohort from a statistical perspective, which required a mininum sample size of 72 to 136 individuals, at a confidence level of 95% and a margin of error of 5%.
Data exclusions	No data were excluded from the analyses
Replication	All conjugation experiments were replicated twice, yielding consistent results.
Randomization	In the conjugation experiment, all hypervirulent Klebsiella pneumoniae isolates carrying the helper plasmids were included, while isolates lacking the helper plasmids within each sequence type were randomly selected. All hypervirulent Klebsiella pneumoniae isolates were conjugated with the same recipient strain and thus randomization is not relevant.
Blinding	Blinding was not feasible, as information regarding the phenotype of experimental isolates was necessary for the selection of transconjugants.

Reporting for specific materials, systems and methods

We require information from authors about some types of materials, experimental systems and methods used in many studies. Here, indicate whether each material, system or method listed is relevant to your study. If you are not sure if a list item applies to your research, read the appropriate section before selecting a response.

Materials & experimental systems

n/a	Involved in the study
☒	☐ Antibodies
☒	☐ Eukaryotic cell lines
☒	☐ Palaeontology and archaeology
☒	☐ Animals and other organisms
☒	☐ Clinical data
☒	☐ Dual use research of concern
☒	☐ Plants

Methods

n/a	Involved in the study
☒	☐ ChIP-seq
☒	☐ Flow cytometry
☒	☐ MRI-based neuroimaging

Plants

Seed stocks

Report on the source of all seed stocks or other plant material used. If applicable, state the seed stock centre and catalogue number. If plant specimens were collected from the field, describe the collection location, date and sampling procedures.

Novel plant genotypes

Describe the methods by which all novel plant genotypes were produced. This includes those generated by transgenic approaches, gene editing, chemical/radiation-based mutagenesis and hybridization. For transgenic lines, describe the transformation method, the number of independent lines analyzed and the generation upon which experiments were performed. For gene-edited lines, describe the editor used, the endogenous sequence targeted for editing, the targeting guide RNA sequence (if applicable) and how the editor was applied.

Authentication

Describe any authentication procedures for each seed stock used or novel genotype generated. Describe any experiments used to assess the effect of a mutation and, where applicable, how potential secondary effects (e.g. second site T-DNA insertions, mosiacism, off-target gene editing) were examined.
